# Supplementary material for: Methylation of DROSHA and DICER as a Biomarker for the Detection of Lung Cancer
Source: Cancers (Basel). 2021 Dec 6;13(23):6139. doi: 10.3390/cancers13236139 (PMC8657200; doi:10.3390/cancers13236139)
Supplement: Supplementary file 1 [file cancers-13-06139-s001.zip › cancers-1459186-supplementary.pdf]

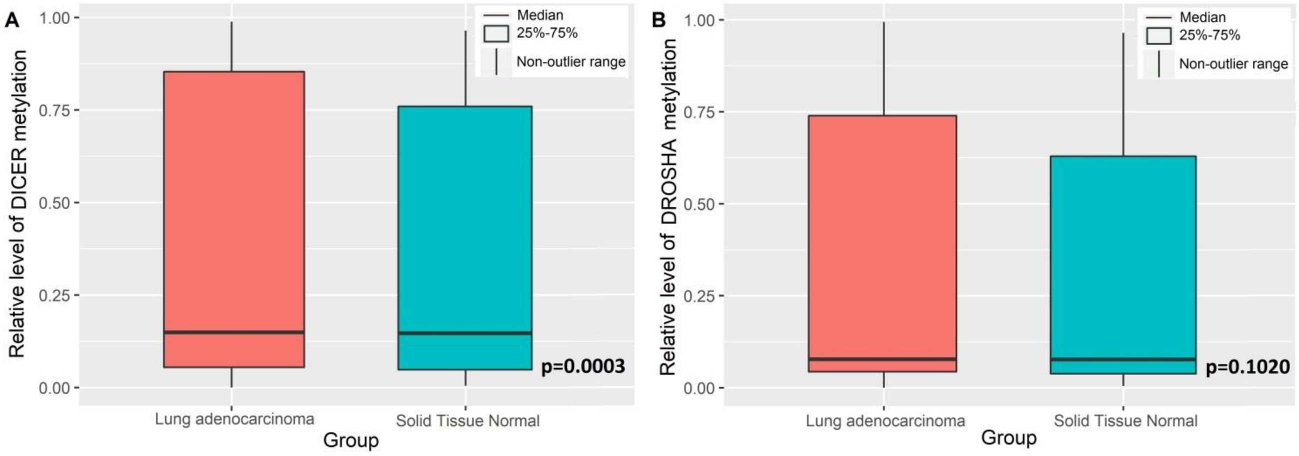

Figure S1. The relative methylation level of *DICER* (A) and *DROSHA* (B) genes in samples from patients with lung adenocarcinoma compared with normal solid tissue (*in silico* analysis of the TCGA data).

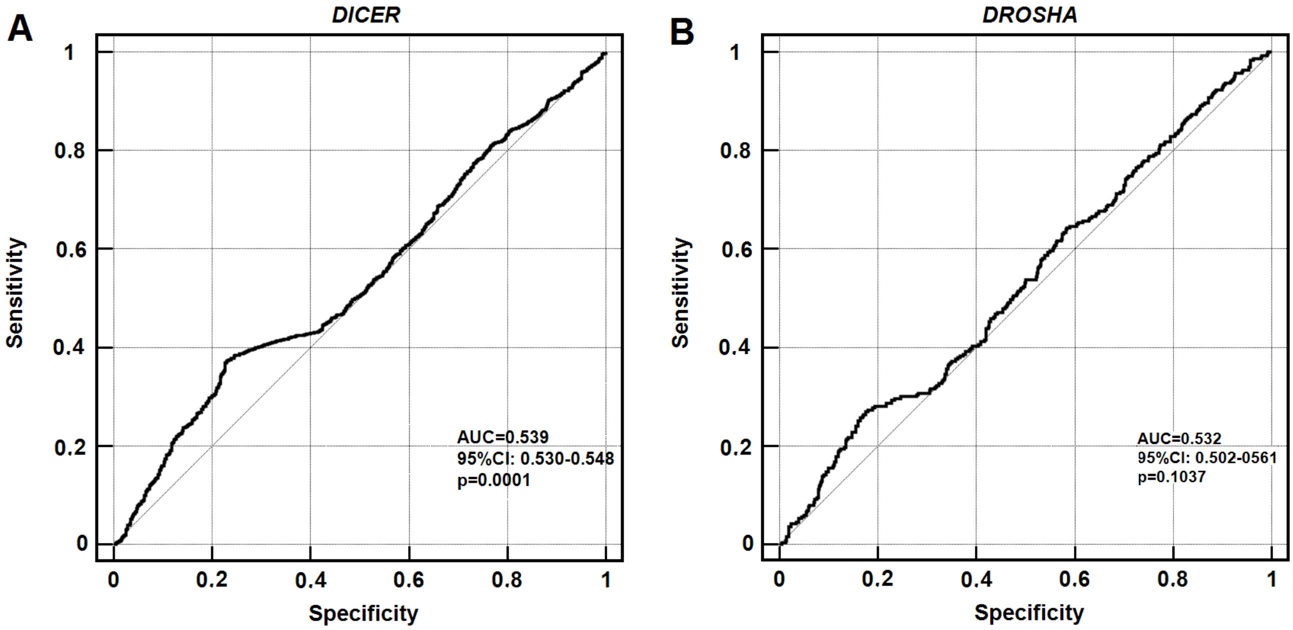

Figure S2. ROC curves analysis to distinguish between normal solid tissue and samples from patients with lung adenocarcinoma on the basis of the relative methylation level of *DICER* (A) and *DROSHA* (B) genes.
